# Management of recurrent apthous ulcer using Corticosteroids, Local anesthetics and nutritional supplements

**DOI:** 10.6026/97320630016992

**Published:** 2020-12-31

**Authors:** Manthra Prathoshini, V Jayanth Kumar, Visalakshi Ramanathan

**Affiliations:** 1 Saveetha Dental College, Saveetha Institute of Medical and Technical Sciences, Chennai, Tamil Nadu, India; 2 Oral Medicine and Radiology, Saveetha Dental College, Saveetha Institute of Medical and Technical Sciences, Chennai, Tamil Nadu, India

**Keywords:** Aphthous ulcer, corticosteroids, local anesthetics, nutritional supplements

## Abstract

Recurrent Aphthous ulcers are the most common oral lesions among dental patients. The ulcers, which usually occur on the nonkeratinized oral mucosa, can cause considerable pain and may interfere with eating, speaking, and swallowing. Therefore, it is of interest to report data on the management of recurrent aphthous ulcer using corticosteroids, local anesthetics and nutritional supplements. Case sheets of 76 patients who underwent treatment for recurrent aphthous ulcer between June 2019 and March 2020 at the Saveetha Dental College, India were used in this analysis. Data was analyzed using Chi square test at a P value < 0.05 that is statistically significant. Results show that topical anaesthetics in population (49%) were more effective that corticosteroids. Thus, topical anaesthetics are recommended for recurrent aphthous ulcer.

## Background

Among the various mucosal diseases, ulcers are the most common type of mucosal disease. The ulcers of the oral cavity due to various reasons and can have varied sizes [1]. The most common ulcers of the oral cavity are the idiopathic apthous ulcers, which are relatively easy to manage. On the other hand ulcers can also occur in response to tobacco usage in the form of a malignancy [2]. Recurrent aphthous ulcers represent a very common but poorly understood oral mucosal lesion. It is spontaneous0, self-limiting ulceration. It occurs in men and women of all ages,gender and geographic regions. The prevalence in Caucasians is in the 3-9% [3]. The cause of recurrent aphthous ulcer is idiopathic in nature. There has also been a viral etiology implicated for apthous recurrent apthous ulcer is classified as minor, major and herpetiform, on the basis of ulcer size and number. Major aphthous ulcers are recurrent, large, chronic, and usually solitary ulcers that begin as nodules, destroy deep tissue, and heal with scarring. It affects the non-keratinised mucosa and posterior mucosal surfaces. Minor aphthous ulcers are recurrent punched-out ulcers, affecting the non-keratinized oral mucosa (lips, buccal mucosa, mucobuccal and mucolabial sulci, and tongue). Herpetiform ulcers are recurrent, multiple, shallow and pinpoint ulcers that may affect any part of the oral mucosa. Major and herpetiform variants are distinctly less common than Minor variant [4]. Behcets disease is a severe variant of Apthous that consists of a triad of oral, genital and ocular lesions [5]. Various factors have been suggested to precipitate outbreaks of recurrent aphthous stomatitis in predisposed persons,including oral trauma, the cessation of smoking for reasons that are unclear [2], anxiety or stress, dietary habits [6], poor oral hygiene [4], sensitivities to food preservatives and agents such as benzoic acid cinnamaldehyde etc. and systemic diseases like Behcet's disease [7]. Treatment of recurrent apthous ulcer remains till date, empirical and nonspecific. Some patients are managed with proper oral hygiene instructions, toothpaste with non-irritant properties and sometimes-palliative therapy for pain [8]. Sometimes natural therapy such as aloe vera was also considered. Patients with history of multiple episodes of recurrent apthous ulcer are given drug therapy. Corticosteroids are traditional treatment of choice for recurrent aphthous ulcer. They are administered as topical gels, mouthwashes and also systemically by oral route. There have been immuno modulators like etanercept also used in the management [9]. Topical anaesthetics are also widely used to relieve pain. [10] In case of nutritional deficiencies, haematological work up including CBC, folate and vitamin B12 are done and replacement therapy such as administration of vitamin B12 supplements is advised. There has also been a role for helicobacter in the role of gastric ulcer management [11]. It is of interest to report the data on the management of recurrent apthous ulcer using corticosteroids, local anesthetics and nutritional supplements.

## Methodology

### Ethical clearance:

The study setting was set in a university setting.Institutional Ethics Committee approval (ethical approval number - SDC/SIHEC/2020/DIASDATA/0619-0320).

### Dataset:

The case records of 76 patients who underwent treatment for recurrent apthous ulcer between June 2019 and April 2020 from Saveetha Dental College is used in this study. The data regarding treatment was divided into 3 groups: topical steroids, topical anaesthetics and nutritional supplements. The inclusion criteria were patientstreated with topical anaesthetics, corticosteroids and nutritional supplements and exclusion criteria were patients treated with antimicrobials.

### Analysis:

The variables recorded were age, gender, therapy and variant. These data were entered into Excel sheet and was cross verified by 2 clinicians. They were obtained using SPSS statistics software and chi-square test was used to determine correlation between gender and recurrent apthous ulcer clinical variants, usage of topical anaesthetics and recurrent apthous ulcer variant, usage of topical corticosteroids and recurrent apthous ulcer variant and usage of nutritional supplements and recurrent apthous ulcer variant. The internal validity of the study was established as the data was collected from a verifiable and standardised database. The external validity is established as the data is from a clinical setup that is duplicatable.

## Results and Discussion:

The data obtained were plotted in the form of bar charts and was analysed. The results showed that, in this study, it was observed that topical anaesthetics was prescribed more frequently for apthous ulcer (49%) among study population and corticosteroids were prescribed least among study population. Figure 1(see PDF) shows clinical variant distribution of apthous ulcer among study population between the gender and minor variant was found to be most common among study population. Figure 2(see PDF) show that usage of topical anaesthetics was high (49%) and Figure 3(see PDF) shows that topical corticosteroids were less prescribed among the study population (23.7%). Figure 4(see PDF) shows that nutritional supplements were equally prescribed among the study population.

Recurrent aphthous ulcers or recurrent aphthous stomatitis is the most common oral mucosal disease among. Despite much clinical and research attention, the causes of Recurrent aphthous ulcer remain a mystery. The ulcers cannot be prevented and the treatment is symptomatic [12]. The recurrent apthous ulcers cause discomfort and pain in patients, affecting their eating habits and quality of their lifestyle [13]. Current treatments mainly used are topical agents such as antimicrobials, amlexanox, anesthetics, and corticosteroids. Other drugs such as azathioprin, colchicine, cyclosporine, thalidomide, should be reserved only in severe cases, as these medications are associated with several side effects when compared to topical medications. Prescription of nutritional supplements also found to have therapeutic effects of lesions [14]. The etiology of the disease is unclear [15]. Salivary genomics has also been extensively applied to arrive at the possible etiological agent for aphthous but with no success till date [16]. In this study, it was observed that topical anaesthetics were prescribed more frequently for aphthous ulcer (49%) and topical corticosteroids were prescribed least among the study population (23.7%). In the clinical variant distribution graph, it was observed that the minor variant was found to be the most prevalent type of variant among the study population. It was observed that topical anaesthetics were prescribed more commonly among the study population. Studies by Altenberg et al. [16] showed that lidocaine in xylocaine, viscous 2% solution can be applied over the lesion for better results.Lidocaine, as a 2% containing gel (Dynexan mouth gel, Gelicaine 2% gel, Xylocaine 2% gel, Lidocaine 2% gel, lidocaine and chamomile extract in Kamistad gel N), or as a spray (Xylocaine pump spray, Xylestesin pump spray, Wick Sulagil throat spray), polidocanol as a paste (Solcoseryl adhesive dental paste), and benzocaine in the form of lozenges (Anaesthesin or Dolo-Dobendan lozenges)are recommended as treatment for Recurrent Aphthous Ulcer. This can be attributed to the fact that patients seek pain relief and want complete recovery from pain within short period of time.

Local anesthetics were used in 49 patients of which 43 are minor, 5 major and 1 herpetiform variant. Local anesthetics were not used in 27 patients of which 26 are minor and 1 major variant. A Chi square analysis was done to compare the usage of local anaesthetics and variants of apthous stomatitis, (chi square 1.623; df-5;p-0.44(P>0.05)) which was not statistically significant due to the disproportionate number of patients in each clinical variant. Topical corticosteroids were prescribed least (23.7%) among the study population. This can be attributed to the fact that long-term use of local steroids may predispose to local candidal infection. This is contradictory to the findings of Barrons et al. [17] and Kerr. A. Ross et al. [18] where their findings showed that topical corticosteroids are the most prescribed medication among patients [19]. This can be due to the fact that there can be an ethnic variation in the population and also in India due to the increases prevalence of Diabetes Mellitus there is always skepticism to use steroids. The increased severity of RAU in HIV-1-infected patients suggests that immunity changes related to this disease may play a role in exacerbating the ulcers. These immune system changes are mainly characterized by low CD4 cell counts. Figure 4(see PDF) showed that nutritional supplements were prescribed equally with topical anaesthetics (38%), but not individually. Previous studies by Brocklehurst P et al. [20] also showed that, many investigators in these studies cited earlier found that high dose replacement therapy with specific vitamins was helpful in the treatment of RAS in patients with documented vitamin deficiencies but that daily multivitamin supplementation, with the RDI of essential vitamins, did not result in a reduction in the number or duration of RAS episodes. No correlation between the type of treatment and the variant of recurrent apthous ulcer was found. Limitations to this study, included limitation to one geographic area and the limited amount of data acquired. However, there have been several studies done in the past from the existing data, which had matched with the already well-established literature data. 9 This study can be utilised to study the effects of each therapy over each variant of apthous ulcer with a larger sample to increase the accuracy of the study.

## Conclusion

Aphthous stomatitis represents the most common ulcer occurring in the oral cavity. We showed that that topical anaesthetics in population (49%) were more effective that corticosteroids. Thus, topical anaesthetics are recommended for recurrent aphthous ulcer. However, it should be noted that a combination therapy of corticosteroids with local anesthetics is effective in the long-term management of aphthous ulcer.
